# A Treatise on Human Physiology; Designed for the Use of Students and Practitioners of Medicine

**Published:** 1872-04

**Authors:** 


### Books Reviewed.

*A Treatise on Human Physiology; designed for the use of students and practitioners of medicine.* By John C. Dalton, M. D., etc. Fifth edition, revised and enlarged. Philadelphia: Henry C. Lea, 1871.

The fifth edition of this truly valuable work on Human Physiology, comes to us with many valuable improvements and additions. As a text book of Physiology the work of Prof. Dalton has long been well known as one of the best which could be placed in the hands of student or practitioner. Prof. Dalton has, in the several editions of his work heretofore published, labored to keep step with the advancement in science, and the last edition shows by its improvements on former ones that he is determined to maintain the high standard of his work.

Space will not permit us to note the additions and improvements made, and indeed, the work has been too long before the profession to need extended notice. We predict for the present edition increased favor, though this work has long been the favorite standard.

